# Internet Gaming Disorder, Problem Gambling Symptoms and Mental Health in Spanish Adolescents: A Cross-Sectional Study on the Role of Microtransactions and Loot Boxes

**DOI:** 10.3390/healthcare14131846

**Published:** 2026-06-25

**Authors:** Juan Manuel Díaz Peña, Richard Kjellgren, Joaquim A. Ferreira, Fernando Fajardo Bullón

**Affiliations:** 1 Programa de Doctorado en Psicología, Facultad de Educación y Psicología, Universidad de Extremadura, Avenida de Elvas s/n, CP 06006 Badajoz, Spain; jdiazpen@alumnos.unex.es; 2 Salvation Army Centre for Addiction Services and Research, School of Public Health and Society, University of Stirling, Stirling FK9 4LA, UK; r.r.kjellgren@stir.ac.uk; 3 Center for Research in Neuropsychology and Cognitive and Behavioral Intervention (CINEICC), Faculty of Psychology and Education Sciences, University of Coimbra, 3004-531 Coimbra, Portugal; jferreira@fpce.uc.pt; 4 Department of Psychology, Facultad de Educación y Psicología, Universidad de Extremadura, Avenida de Elvas s/n, CP 06006, Badajoz, Spain; 5 Instituto Universitario de Investigación y Prospección Educativa-INPEX, Universidad de Extremadura, CP 06006 Badajoz, Spain

**Keywords:** adolescents, internet gaming disorder, gambling, emotional well-being, loot boxes, microtransactions, behavioral addictions, academic performance

## Abstract

**Background/Objectives**: Adolescent mental health problems have increased in recent years, with growing concern about the impact of digital behaviors such as problematic video game use and gambling. Internet Gaming Disorder (IGD) and Problem Gambling Symptoms may share psychological risk markers, but evidence in Spanish adolescents is limited. This study aimed to examine the relationship between IGD, problem gambling symptoms, and mental health, and to identify sociodemographic, psychological, and behavioral factors associated, including microtransactions and loot boxes. **Methods**: A cross-sectional study was conducted with secondary education students from Extremadura (Spain). The final sample included 343 participants. Measures included an ad hoc questionnaire on video game use, the IGDS9-SF, SOGS-RA, and the Strengths and Difficulties Questionnaire (SDQ). Descriptive analyses, Spearman correlations, and multivariable regression (Poisson and negative binomial) were performed. Results: IGD and gambling were positively correlated (Spearman’s *ρ* = 0.386, *p* < 0.001) and associated with higher mental health difficulty scores (IGD: *ρ* = 0.299, *p* < 0.001; gambling: *ρ* = 0.214, *p* < 0.001). Male gender was associated with both outcomes (IGD: incidence rate ratio [*IRR*] = 1.21 [95% Confidence Interval: 1.13–1.30]; gambling: *IRR* = 2.90 [1.85–4.60]). Microtransactions were associated with both behaviors (IGD: *IRR* = 1.17 [1.09–1.25]; gambling: *IRR* = 1.74 [1.19–2.54]), while loot box use was related only to IGD (IRR = 1.13 [1.05–1.21]). Total SDQ score was positively associated with both IGD (*IRR* = 1.02 [1.02–1.03]) and gambling (*IRR* = 1.10 [1.06–1.13]). Younger age was associated with higher IGD scores (*IRR* = 0.97 [0.96–0.99]). **Conclusions**: There are similarities in the associations among the examined factors and increased scores of IGD and gambling in adolescents, particularly male gender, higher mental health difficulties scores, and involvement in monetized gaming systems. School-based, family, and public health prevention strategies may benefit from addressing the importance of psychological well-being and increase awareness of the potential risks associated with digital gaming practices.

## 1. Introduction

Mental health care is an essential protective factor for the overall well-being of children worldwide, with particular relevance during adolescence, a critical developmental stage in which cognitive, social, and emotional skills are acquired and consolidated [1]. The World Health Organization (WHO) defines mental health as a state of well-being in which individuals are aware of their own abilities, can cope with normal life stress, work productively, and contribute to their community. It is not merely the absence of disease, but a state of balance. According to the WHO, more than 13% of adolescents aged 10–19 live with a mental disorder, representing approximately 89 million boys and 77 million girls worldwide, with anxiety and depression accounting for around 40% of cases [2]. In Europe, nearly 9 million adolescents aged 10–19 have a diagnosed mental disorder [3], with Spain showing one of the highest prevalence rates of mental health problems among adolescents, particularly behavioral and emotional disorders [4].

Research also indicates that sex and age influence the expression of emotional difficulties, with girls more likely to report higher levels of anxiety and depressive symptoms, whereas boys tend to show higher levels of behavioral problems and hyperactivity [5].

In Spain, mental health difficulties have been negatively associated with academic performance, especially emotional problems among students with access to digital resources [6]. Post-lockdown studies have reported that stress, impulsivity, and time spent online are directly related to Internet Gaming Disorder (IGD), with mental health being the psychosocial factor most strongly associated with problematic use among adolescents [7]. Similarly, research conducted in a rural educational context found that greater time spent playing video games was related to poorer mental health in students [8].

However, these findings differ from those reported in the Power of Play: Global Video game Report [9], which, based on international survey data, highlights social and emotional benefits of video game use, such as reduced stress, lower anxiety, decreased loneliness, and improvements in creativity, problem-solving, adaptability, and teamwork, suggesting that video games may function not only as a consequence but also as a coping resource for emotional difficulties. However, the industry-based nature of this source should be considered when interpreting its findings, as it may introduce potential commercial bias. This highlights the need for additional independent scientific studies to generate more objective evidence regarding the complex relationship between video game use and psychological well-being.

Current research trends indicate that youth mental health faces increasing challenges related to school, family, and social contexts, as well as emerging issues linked to technology use, including social media and video games [10]. Although substantial research has examined mental health, problematic gaming, and gambling separately, fewer studies have investigated these phenomena simultaneously in adolescent populations. Evidence is particularly limited regarding the role of monetized gaming features, such as microtransactions and loot boxes, and their associations with both IGD and gambling-related problems. Important gaps remain, particularly regarding how mental health influences problematic video game use [11], and the need for studies integrating measures of new technology use and addictive behaviors (video games, online gambling), together with further exploratory studies to better understand contextual factors and inform prevention efforts in diverse settings [12].

### 1.1. Video Game Use and Gambling

In recent decades, the addictive potential of video games has been widely debated, leading to the inclusion of Internet Gaming Disorder (IGD) in both the DSM-5 and the ICD-11. This disorder is defined as recurrent and persistent engagement in video games (online and offline) for many hours, resulting in significant impairment over a period of at least 12 months [13].

According to data from WHO [14], the global prevalence of IGD in adolescents is estimated at 8.6%. In Europe, around 12% of adolescents are considered “at risk of problematic gaming”, with higher risk in boys (16%) than in girls (7%).

In Spain, as part of the National Strategy on Addictions, a video game module was included in 2019 in the national survey of secondary school students on drugs and addictions. In the 2023 survey, 83.1% of students reported having played video games, with this activity being much more frequent among boys. Regarding age, a decrease in frequency of use is observed from 14 to 17 years, followed by an increase at age 18. Overall, 5.1% of students aged 14 to 18 would present a possible video game use disorder, spending more than 8 h per day playing video games [15].

Problematic video game use among Spanish adolescents is associated with multiple factors, including poor impulse control [16]. The central role of obsessive passion has been highlighted as a key predictor, showing a significant association between online gaming and increased IGD [16]. IGD severity is negatively correlated with quality of life and with adolescents’ psychological and emotional well-being [10,17,18]. Furthermore, a recent systematic review of the literature of adolescents with problematic gaming highlighted substantial psychiatric comorbidity, particularly with behavioral or emotional difficulties. The review also suggests that these comorbidities may interact bidirectionally with gaming problems across development, reinforcing the need for studies that examine adolescent mental health in different contexts [19].

Pathological gambling (problem gambling symptoms), classified in the DSM-5 as an addictive disorder (rather than an impulse-control disorder), is defined as a persistent and recurrent maladaptive gambling behavior that leads to clinically significant impairment or distress, with serious personal, family, and financial consequences [20].

At the global level, a recent meta-analysis [21] estimates that among adolescents who gambled in the past year, the prevalence of problematic gambling reaches approximately 13%. In Europe, data from the European School Survey Project on Alcohol and Other Drugs [22] show that 23% of European students aged 15–16 have gambled money in the past year, and about 9% of gamblers present a problematic gambling profile.

In Spain, 21.5% of students aged 14 to 18 reported online and/or in-person gambling in the last 12 months. Significant sex differences were found, with this percentage rising to 29.4% in males and decreasing to 13.3% in females [15]. However, other studies report results in which females aged 13–14 score higher (65%) than males in online gambling addiction [23]. Therefore, further analysis of the impact of sex on gambling behavior appears necessary.

The age of onset for online and in-person gambling is similar (14.7 and 14.8 years, respectively). Regarding the type of activity, video games rank first, with more than half of young people admitting having gambled money within video games (51.5%), followed by sports betting [15].

Online gambling among Spanish adolescents has received increasing attention due to its rapid expansion and accessibility through mobile devices [24]. Several systematic reviews document significant participation rates and variable levels of problematic risk; moreover, online gambling is associated with greater psychiatric comorbidity, including depressive and anxious symptoms, impulsive behavior, and substance use in some subgroups [24,25].

Gambling in adolescents is a relevant and emerging problem [24,25]. Some studies report that the correlation between problematic video game use and problem gambling symptoms is not uncommon [26], and both share risk markers such as poor emotional regulation and deficits in impulse control. In addition, the negative impact on mental health and psychosocial functioning is particularly pronounced in individuals scoring high in both disorders.

### 1.2. Relationship Between Mental Health, Video Games and Gambling

Although the relationship between mental health and the use of video games or gambling is still unclear, it is even more uncertain whether these activities may have beneficial or harmful effects during adolescence.

A systematic review reports several longitudinal studies in which pre-existing psychopathology predicts the later development of IGD [25]. Other studies show positive correlations between high IGD scores and greater emotional and behavioral difficulties [26]. However, evidence suggests that IGD behaviors are often symptoms or manifestations of underlying mental health problems, rather than direct causes, indicating bidirectional relationships [27]. In addition, IGD shows positive correlations with depression scales and childhood traumatic events, while psychological resilience moderates this effect and acts as a protective factor [28,29]. This relationship has not yet been analyzed in Spanish populations, and it is necessary to determine whether these correlations also appear in other countries.

Regarding problem gambling symptoms, research with adolescents shows significant correlations with mental health problems such as impulsivity, emotional distress, and psychiatric symptomatology [29]. Gambling is also frequently associated with deficits in emotional regulation, behavioral problems, and low academic performance [28,30,31]. Gambling may also be represented in video games through loot boxes, which are mechanisms integrated into many games through which users can obtain random rewards in exchange for payment, either with real money or with virtual in-game currency created by the game itself [32]. In contrast, microtransactions refer to any digital purchase made within a video game to obtain special items, additional downloadable content, or virtual currency used within the game, without involving chance-based mechanics.

Jiménez-Murcia et al. [33] show that the use of loot boxes may act as a mediating factor between gaming and gambling. Specifically, research conducted by Wilson et al. [34] confirms that individuals who purchase loot boxes are almost three times more likely to suffer adverse financial consequences and experience psychological distress, even when controlling for other variables such as gambling. According to other authors, there is a correlation between IGD and gambling in which digital purchases and loot boxes act as the link, revealing a 5.6-fold increased probability of developing problematic gambling among individuals with problematic video game use [26,35]. Several studies highlight the need for further research examining the possible transition from loot box use to problem gambling symptoms and the development of IGD in Spanish adolescents [32,36,37,38].

This study contributes to the literature by examining whether IGD and gambling-related problems in Spanish adolescents share common psychological and behavioral correlates, and by testing whether monetized gaming practices show similar or distinct associations with these two outcomes.

### 1.3. Current Study

The present study is an exploratory study aimed at examining shared risk markers associated with problematic behaviors. The aim of the study is to analyze the relationship between Internet Gaming Disorder (IGD), Problem Gambling Symptoms, and mental health in a Spanish adolescent population. In addition to examining this relationship, the study seeks to identify the variables that are associated with IGD and Problem Gambling Symptoms according to sociodemographic factors (such as sex, age), use of microtransactions, loot boxes, and mental health difficulties.

Research hypotheses.

**H1.**  *There are significant correlations between mental health difficulties, IGD, and Problem Gambling Symptoms.*

**H2.**  *Boys will obtain higher scores in IGD and Problem Gambling Symptoms than girls.*

**H3.**  *Higher levels of mental health difficulties will be associated with higher IGD and Gambling Symptoms scores.*

**H4.**  *Older students will show higher scores in IGD and Problem Gambling Symptoms.*

**H5.**  *The use of microtransactions and loot boxes are associated with IGD and Problem Gambling Symptoms.*

## 2. Methodology

### 2.1. Participants

A sample of 850 students from Secondary Education (ESO), Baccalaureate, was selected, including 430 females (50.5%), 404 males (47.5%), and 16 individuals who preferred not to specify their gender (1.9%), with a mean age of 14.28 years (*SD* = 1.45). The participants came from nine educational centers located in the provinces of Badajoz, Cáceres and Mérida (Extremadura, Spain). A two-stage cluster sampling method was used for participant selection. Of the total sample, 57.6% of students were from rural settings, while 42.4% were from urban areas. Regarding grade level, 25.9% were in 1st year of ESO, 13.9% in 2nd year, 28.9% in 3rd year, 25.6% in 4th year, and 5.6% in 1st year of Baccalaureate. All surveyed students completed the questionnaire satisfactorily.

**Figure 1:**
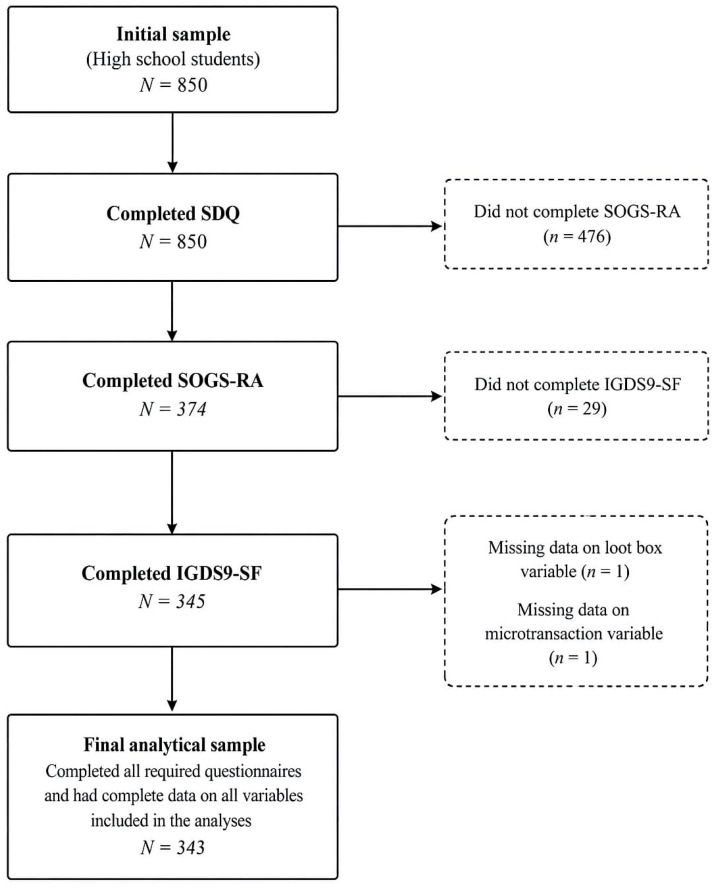
Flow diagram.

The final sample consisted of 343 students. The reduction in the sample is primarily due to the use of filter questions in the survey design. Participants who reported not engaging in gambling or video game activities, in a previous question, were not administered the corresponding questionnaires (SOGS-RA and IGDS9-SF), whereas those who reported engaging in these activities proceeded to complete the relevant measures. A total of 476 cases had non-completion data on SOGS-RA (ranging from 422 to 447 across the 12 SOGS-RA questions). After excluding the 476 cases with non-completion data on SOGS-RA, 374 cases remained. In total, 29 of the remaining cases had non-completion data for the IGDS9-SF measure and were excluded, leaving the sample size at 345. One participant was excluded due to missing data on the loot box variable, and one participant was excluded due to missing data on the microtransaction variable, meaning the final sample size was 343 cases. The authors did not consider it appropriate to use multiple imputations due to the large number of participants who did not complete the SOGS-RA, which is one out of the two outcome variables. We used listwise deletion, and caution needs to be exercised in interpreting the findings as a result of this. The final sample selection process is illustrated in Figure 1. The characteristics of the final sample are described in Table 1 and a comparison of the final and original samples across outcome and associated variables is provided in Supplementary Materials.

### 2.2. Instruments

First, an ad hoc questionnaire was developed to collect data related to video game and loot box use. This questionnaire included a brief introductory text providing explanatory information (These *questions ask about your video game playing activity during the last year* [i.e., *the last 12 months]. By video game playing, we mean any activity related to playing video games, whether you played on a computer, video game console, or any other type of device* [e.g., *cell phone, tablet*, etc.], *online or offline*). The introductory text was followed by specific questions aimed at obtaining key information, such as whether they played video games (*Yes/No, If your answer was No, please proceed to the next questionnaire*). To measure the variable *“Multiplayer video game type”*, participants were asked: “*What type of video games do you usually play? (Action, adventure, sports, open world, multiplayer, puzzle*, etc.)”. To assess *frequency of use*, the question was “*How many hours do you spend playing video games per week?*”, with options ranging from less than 1 h to more than 20 h in progressive intervals. To measure *loot box use*, we included the following introductory text prior to the questionnaire items (“*Loot boxes can be boxes, chest, envelopes, gashapon, or virtual piñatas that contain random items from a videogame, allowing you to obtain virtual items such as characters, objects, weapons, or skins [appearances]*”), the following question was included: “*Have you ever purchased any loot boxes in the past year?*” (Yes/No) and to measure microtransactions, we also included another introductory text (“*Microtransactions are small digital purchases to obtain digital content, in-game currency, or subscriptions*”)*, the following question was included: “Have you made any microtransactions in the past year?*” (*Yes/No*). The inclusion of these items responds to the lack of specific instruments that comprehensively address these aspects, allowing for a more complete and accurate evaluation of current gaming habits.

The Strengths and Difficulties Questionnaire (SDQ) was used as a tool to identify potential emotional and behavioral difficulties during adolescence. According to Boyer et al. (2016) [39], this instrument shows high validity and effectiveness in detecting psychiatric disorders in the general population. Translated into more than 66 languages, it has been widely used internationally and in several Spanish studies [40,41], representing an improvement over traditional tools such as Achenbach’s *Child Behaviour checklist (CBLC)* [40]. Different versions of the SDQ exist for educational, psychological, and clinical contexts, which, although slightly different, share the same basic structure [42]. The questionnaire consists of 25 items grouped into five dimensions: conduct problems, emotional symptoms, hyperactivity, peer problems, and prosocial behavior. Of these items, 20 are used to calculate the total difficulty score. Internal reliability in the current study, assessed by Cronbach’s Alpha, was found to be acceptable (*α* = 0.78 [95% confidence intervals: 0.74–0.81]). Due to methodological considerations, the authors used the total difficulties score as a measure of overall mental health difficulties, with scores ranging from 0 to 40 points.

To evaluate the variable Problematic Video Game Use (Gamer), the IGDS9-SF [43] was employed. This scale was designed to assess the intensity and consequences of *Internet Gaming Disorder (IGD)*. It comprises nine items exploring problematic video game use, both online and offline, during the past year. Responses are rated on a five-point Likert scale, yielding scores between 9 and 45 points, where higher scores indicate greater IGD severity. Although the instrument does not provide a definitive diagnosis, it estimates the severity of the problem; a cut-off score of 36 points is established as the diagnostic threshold, as proposed by Sánchez-Iglesias et al. [44] in their Spanish validation study.

The systematic review by Poon et al. [45] concluded that the IGDS9-SF demonstrates adequate internal consistency, excellent criterion validity, a well-defined unidimensional structure, and strong evidence of measurement invariance across sex and age groups. These findings support its reliability for assessing IGD. Internal reliability in the current study, assessed by Cronbach’s Alpha, was found to be acceptable (*α* = 0.78 [95% confidence intervals: 0.74–0.81]).

To assess Problem gambling symptoms, the SOGS-RA [46] was used. This is a youth adaptation of the South Oaks Gambling Screen [47], specifically designed to evaluate problem and at-risk gambling among Spanish adolescents. The questionnaire consists of 12 dichotomous (Yes/No) items. The SOGS-RA provides three categories: non-gambler/no problems, at-risk gambler, and problem gambler. A non-gambler or no-problem gambler is one who answers affirmatively to none or only one item; an at-risk gambler answers affirmatively to 2–3 items; and a problem gambler to 4 or more items. Problem gamblers experience significant issues that may negatively impact daily functioning (e.g., academic performance, relationships with parents and friends), while at-risk gamblers are in a less severe state but may already be facing or developing gambling-related problems. The Cronbach’s Alpha confirmed internal reliability to be acceptable (*α* = 0.74 [0.70–0.78]).

### 2.3. Procedure

A two-stage cluster sampling procedure was employed. First, the territory of Extremadura was divided into six geographical clusters based on the territorial area established by the Regional Ministry of Education (Badajoz, Mérida, Cáceres, Plasencia, Don Benito-Villanueva de la Serena, and Llerena-Zafra) to ensure adequate representation of the region’s educational diversity. Four clusters were then randomly selected: Badajoz, Don Benito-Villanueva de la Serena, Cáceres and Mérida.

In the second stage, nine schools were randomly selected from the chosen clusters. If a school contacted declined to participate, it was replaced by another school randomly selected from the same cluster. Of all the schools contacted, nine agreed to participate in the study, resulting in representation from each of the four selected clusters.

After coordinating with each school’s Guidance Department, questionnaires were distributed via online links for digital completion. Prior to administration, informed consent was obtained from parents, students, and teachers, and they were informed of the study’s objectives. Participants were assured of the confidentiality of the collected data and that it would be used solely for research purposes.

Questionnaires were completed online using tablets or computers during students’ tutorial sessions, with an approximate duration of 30 min. Tutors received prior instructions from the research team to assist with any questions during the process.

The study received ethical approval from the Bioethics Committee of the University of Extremadura, registration number 168//2025.

### 2.4. Statistical Analysis

We had two dependent variables: problematic video game use (IGDS9-SF) and problem gambling symptoms (SOGS-RA). The final analysis is based on a subset of the surveyed students which had answered questions from the IGDS9-SF and SOGS-RA instruments and had complete data across all variables used in the analysis (*n* = 343). As such, no imputation of non-completion data was required. Descriptive statistics for the final sample are provided in Table 1. Data cleaning, processing and analysis were performed in R version 4.4.2 (https://www.R-project.org/).

Our analysis begins with a presentation of descriptive statistics for key demographic variables, covariates and dependent variables. This is followed by visual representations of the distributions of the two dependent variables. We then examine Spearman’s rank correlation coefficients to quantify the relationships among IGDS9-SF, SOGS-RA and total SDQ score. Finally, we present the results from two multivariable regression models.

**Figure 2:**
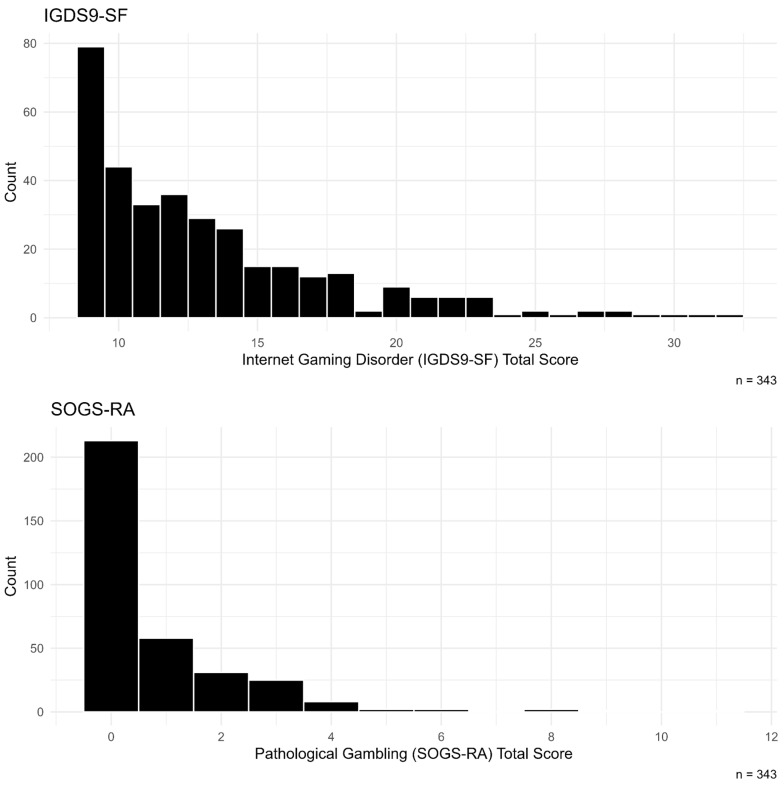
Distribution of outcome variables.

Table 2 shows summary statistics for the two outcome variables, which suggests issues with overdispersion (i.e., variance > mean) and Figure 2, showing the distributions visually, shows that both distributions are right skewed. A series of statistical tests were performed to guide model selection. Shapiro–Wilk tests confirmed that both the IGDS9-SF (*W* = 0.835, *p* < 0.001) and SOGS-RA (*W* = 0.6155, *p* < 0.001) did not follow a normal distribution, and thus unsuitable for linear regression. We fitted both outcomes as Poisson models (with covariates) and formally checked for overdispersion using the methods of Gelman and Hill [48], and a significant degree of overdispersion was identified in the SOGS-RA model (dispersion ratio = 1.83, *Pearson’s Chi-Squared* = 614.15, *p* < 0.001), but not in the IGDS9-SF model (dispersion ratio = 0.960, *Pearson’s Chi-Squared* = 322.72, *p* = 0.689). We therefore elected to use a Poisson model for IGDS9-SF. The Poisson model offered a pragmatic approach to model the skewed summed scores, but we also considered alternative modelling approaches and checked the substantive findings against other models. We conducted a sensitivity analysis to compare the Poisson estimates to estimates from an ordinal logistic regression model. Both models led to highly similar substantive findings, with very similar coefficients in terms of the significance and direction of main effects. The Akaike information criterion was lower for the ordinal model (*AIC* = 1631.068), compared to the Poisson model (*AIC* = 1819.922), but the R2 Nagelkerke was much higher for Poisson (0.546) compared to the ordinal model (0.358). Whereas the original questions underlying the IGDS9-SF are on an ordinal Likert type scale, modelling it as an ordinal variable in ordinal logistic regression would ideally collapse the response values into fewer ordinal categories (e.g., very low, low, medium, high, very high), which would be preferable if there was a strong theoretical rationale for such an operationalization; however, it is not ideal due to the fact that it reduces the amount of information available. We therefore chose to use Poisson because the outcome is a multi-item summed score with a wide range of values, and the choice of the Poisson model was guided by the distributional properties of the aggregate score, and that count models has previously been used to model psychometric outcomes [49,50].

For the SOGS-RA outcome, the identified issue of overdispersion suggested a negative binomial may be more appropriate. We therefore used SOGS-RA as the outcome in a negative binomial and again assessed overdispersion: no significant degree of overdispersion was identified (*dispersion ratio* = 0.759, *p* = 0.560). However, it is clear from Table 2 and Figure 2 that there is an excess of zeros in the SOGS-RA variable. We checked the negative binomial model for zero inflation, and the model predicted 208 zeros, whereas 213 zeros were observed (ratio = 0.98), and there was not a significant degree of zero inflation (*p* = 0.584). Nevertheless, we conducted a sensitivity analysis comparing the negative binomial model with several iterations of zero-inflated negative binomial models (with variable combinations in the count and zero-inflated parts of the model), and the estimates were very similar in terms of the patterning of significance, magnitude and direction of main effects. AIC (ranging from 791.642 to 794.662) and *R2 Nagelkerke* (ranging from 0.308 to 0.361) statistics were very similar across the models, and we chose to report the estimates original negative binomial model (*AIC* = 792.447, *R2 Nagelkerke* = 0.337) since we did not have a strong theoretical rationale for using a zero-inflated model specification, which is appropriate in situations where there are parallel data generating processes leading to an excess of zeros on the one hand, and positive counts on the other [51].

Estimates are exponentiated and presented as incidence rate ratios (IRRs) with 95% confidence intervals. We used the same set of covariates for both models to allow for comparability (Table 3). An assessment of multicollinearity confirmed low correlations among associated variables in both models with variance inflation factor scores ranging from 1.01 for age to 1.32 for gender in the IGDS9-SF model, and, similarly, 1.02 for age and 1.30 for gender in the SOGS-RA model.

## 3. Results

The sample characteristics are presented in Table 1. Most participants were male (61%), had a mean age of 14.24 (*SD* = 1.49). In total, 22% had opened a loot box in the last year, and 35% had used microtransactions in the last year.

The distributions of the two outcome variables are presented in Figure 2. It can be observed that they are both right-skewed. The mean IGDS9-SF score was 13.2 (*SD* = 4.6), and 0.83 (*SD* = 1.48) for SOGS-RA.

We calculated pairwise Spearman’s rank-order correlations among SOGS-RA, IGDS9-SF and total SDQ score. This showed that all three associations were positive and statistically significant. IGDS9-SF was moderately positively correlated with SOGS-RA (*Spearman’s ρ* = 0.386, *S* = 4,205,493, *p* < 0.001), and with SDQ (*ρ* = 0.299, *S* = 31,632,132, *p* < 0.001). SOGS-RA was positively correlated with SDQ (*ρ* = 0.214, *S* = 6,852,443, *p* < 0.001).

The results from the statistical models in Table 4 show that there are significant differences in gender for both problem gambling symptoms and problematic video game use. Males have significantly higher scores compared to females for both problem gambling symptoms (*Incidence Rate Ratio* = 2.89 [95% Confidence Interval: 1.84–4.59]) and problematic video game use (*IRR* = 1.21) [1.13–1.30]). The association is much stronger for problem gambling symptoms, and the association is comparatively weak for problematic video game use (*IRR* = 0.97) [0.96–0.99]). There is no association between age and problem gambling symptoms when controlling for other factors; however, age is negatively associated with problematic video game use (*IRR* = 0.978 [0.96–0.99]). There is a small, but positive association between having opened a loot box in the previous year and problematic video game use (*IRR* = 1.13 [1.05–1.21]), but there is no statistically significant association between loot boxes and problem gambling symptoms. However, having used microtransactions in the last year is positively associated with both problem gambling symptoms (*IRR* = 1.74 [1.19–2.54]) and problematic video game use (*IRR* = 1.17 [1.09–1.25]). Total SDQ score is associated with both problematic video game use (*IRR* = 1.02 [1.02–1.03]) and problem gambling symptoms (*IRR* = 1.10 [1.06–1.13]), though the magnitude of the associations is modest.

Problem gambling symptoms and problematic video game use are correlated, and the statistical models also confirm that the risk marker with problem gambling symptoms and problematic video game use are highly similar. For problem gambling symptoms, being male, using microtransactions and an increased SDQ score are potential risk markers. Similarly, being male, opening loot boxes, using microtransactions, and higher SDQ scores were associated with problematic video game use. In addition, the results suggest higher levels of problematic use among younger students compared with their older peers. Our results are limited by a small sample but random sample. Our study nevertheless provides important evidence which suggests that problematic video game use and problem gambling symptoms are correlated, and that they share similarities in terms of risk markers (see Table 4).

## 4. Discussion

The main objective of this study was to analyze the relationship between problematic video game use (IGD), gambling, and mental health in a Spanish adolescent population, and by testing whether monetized gaming practices show similar or distinct associations with these two outcomes. Overall, the results confirm the existence of a significant relationship between IGD, gambling, and mental health difficulties, showing a partially shared pattern of risk markers, particularly, male sex and the use of microtransactions.

H1 is supported. Positive and statistically significant associations were observed among IGD, gambling symptoms, and overall mental health difficulties measured through the SDQ. The strongest association was found between IGD and gambling symptoms (*ρ* = 0.386), followed by the association between IGD and SDQ total score (*ρ* = 0.299), whereas the association between gambling symptoms and SDQ total score was weaker, although still statistically significant (*ρ* = 0.214). These findings are consistent with the previous literature reporting significant relationships between mental health difficulties, problematic gaming, and gambling-related behaviors among adolescents [27,28,29,30,31]. Previous studies have highlighted that adolescents presenting greater psychological distress tend to report higher levels of problematic gaming and gambling symptoms, although the directionality of these relationships remains unclear [27,28]. Likewise, the positive association observed between IGD and gambling symptoms is consistent with studies suggesting that both behaviours share common psychological and behavioural mechanisms, including impulsivity, reward sensitivity, and maladaptive coping strategies [18,40]. The fact that the strongest association was observed between IGD and gambling symptoms provides further support for the notion that these behaviours may be part of a broader continuum of digital risk behaviours [40]. Although the magnitude of the correlation was moderate, the findings suggest that adolescents reporting higher levels of one behaviour also tend to report higher levels of the other.

H2 is supported. Male obtained significantly higher scores than female in both, problematic video game use (*IRR* = 1.21) and gambling symptoms (*IRR* = 2.89), after controlling for sociodemographic, academic, and behavioural variables. The association was considerably stronger for gambling symptoms than for IGD. These findings are consistent with previous European and Spanish studies reporting greater involvement of male adolescents in both problematic gaming and gambling activities [16,17]. Several explanations have been proposed in the literature, including a greater preference among boys for competitive online games, higher exposure to gaming environments incorporating monetization systems, and greater engagement in risk-taking behaviours [52]. However, recent evidence has suggested that some forms of online gambling may be increasing among adolescent girls in specific contexts [24], indicating that gender differences should continue to be monitored as digital gaming and gambling environments evolve.

H3 is supported. Higher total SDQ scores were significantly associated with higher levels of both problematic video game use (*IRR* = 1.02) and gambling symptoms (*IRR* = 1.10), even after adjustment for demographic, academic, and gaming-related variables. Although the magnitude of the associations was modest, the results suggest that adolescents reporting greater overall emotional and behavioral difficulties also tended to report higher levels of both behavioural outcomes. These findings are consistent with previous research linking poorer mental health with problematic gaming and gambling-related behaviours [7,26,30]. Rather than specific emotional or behavioural domains alone, the present findings suggest that overall psychosocial difficulties may be relevant when examining adolescent vulnerability to behavioural addictions. This interpretation is in line with studies reporting that psychological distress, emotional dysregulation, and behavioural difficulties frequently co-occur with problematic engagement in gaming and gambling activities [27,30]. Given the cross-sectional nature of the study, the findings do not allow conclusions regarding causality. However, they reinforce previous evidence suggesting that mental health difficulties and behavioural addictions are closely interconnected during adolescence and should be considered jointly in prevention and intervention initiatives.

H4 is not supported. Age was not significantly associated with gambling symptoms after controlling for other variables. In contrast, age showed a small negative association with problematic video game use (*IRR* = 0.97), indicating that younger students tended to report slightly higher IGD scores than older students. This finding differs from the original hypothesis and from some epidemiological reports suggesting increased gambling and gaming involvement during later adolescence [16]. However, it is broadly consistent with evidence indicating that intensive engagement with video games often peaks during early and middle adolescence before declining as leisure interests diversify [16]. It is also important to consider the characteristics of the present sample, where the mean age was 14.24 years and relatively few participants were represented at the upper end of adolescence. Therefore, the observed association should be interpreted cautiously. Other variables not included in the present analyses, such as parental supervision, family environment, peer influence, or gaming time, may partly explain age-related differences reported in previous studies [53].

H5 is largely supported. The use of microtransactions was positively associated with both problematic video game use (*IRR* = 1.17) and gambling symptoms (*IRR* = 1.74). In contrast, opening loot boxes was positively associated with problematic video game use (*IRR* = 1.13) but was not significantly associated with gambling symptoms after statistical adjustment. These findings are broadly consistent with previous studies highlighting the role of in-game spending systems as an important correlate of problematic gaming and gambling-related behaviours [35,36,54]. Microtransactions emerged as the behavioural variable most consistently associated with both outcomes. This finding may reflect the increasing normalisation of spending money within digital gaming environments and the growing integration of gambling-like mechanics into contemporary video games [9].

The absence of a statistically significant association between loot box use and gambling symptoms differs from some previous studies proposing that loot boxes may act as a bridge between gaming and gambling behaviours [32,36]. However, it is possible that in the present sample, loot box engagement is more closely related to gaming involvement itself than to gambling-related behaviours. Another possible explanation relates to the measurement of loot box engagement in the present study. Loot box use was assessed through a dichotomous item asking whether participants had opened loot boxes during the previous year, without capturing the frequency of purchases, expenditure, or intensity of engagement. Previous research has suggested that gambling-related harms are more strongly associated with frequent or high-spending loot box use than with simple exposure [35,36]. Furthermore, the relatively young age of the sample should be considered when interpreting gambling-related findings. Since access to most forms of legal gambling in Spain is restricted to adults, younger adolescents may have had fewer opportunities to engage in gambling activities than participants included in older adolescent or young adult samples. Consequently, the absence of a statistically significant association with gambling symptoms should be interpreted cautiously.

Overall, the findings suggest that problematic video game use and gambling symptoms share several common correlates, particularly male gender, microtransaction use, and poorer overall mental health. These results highlight the importance of incorporating mental health promotion and critical digital literacy into prevention programs aimed at adolescents. Social policies should address the importance of psychological well-being and increase awareness of the potential risks associated with digital gaming practices.

Among the strengths of the present study is the use of validated instruments (IGDS9-SF, SOGS-RA, SDQ), which have high acceptance within the scientific community according to the evidence-based clinical guideline on behavioral addictions of the Spanish National Drug Plan [55], as well as statistical modeling appropriate to the data distribution. Several limitations should be considered when interpreting the findings of this study. First, the sample was drawn exclusively from the Spanish region of Extremadura, which may limit the generalizability of the results to adolescents from other regions or countries. This is an exploratory study and was not intended to obtain a representative sample of the adolescent population in Extremadura. Second, all measures relied on self-report questionnaires and are therefore susceptible to common sources of bias, including recall error and social desirability effects. This issue may be particularly relevant for gambling-related behaviors, as underage participants may have been reluctant to disclose involvement in activities that are legally restricted, potentially leading to underreporting. Furthermore, because the main analyses required complete data across all assessment instruments, the final sample included in the analyses was reduced to 343 participants. Although this restriction ensured methodological consistency across variables, it may also have introduced selection bias, including potential differential attrition by sex. In addition, loot box purchases and microtransactions were assessed using dichotomous (yes/no) items. Although this approach facilitated data collection, it did not capture important dimensions such as frequency, expenditure, or intensity of engagement, which would allow for a more nuanced understanding of these behaviors. Additionally, as this was an anonymous exploratory study, no support or referral information was provided. Future research on behavioral addictions and gambling-related problems may consider providing participants with information on available support resources. Finally, the study did not account for potential contextual factors, such as family characteristics or socioeconomic status. Future research should address these limitations by incorporating more diverse samples, multi-method assessment strategies, and a broader range of individual and contextual variables.

## 5. Conclusions

In conclusion, problematic video game use and problem gambling symptoms were positively associated in this sample of Spanish adolescents and showed similar patterns of association with male gender, overall mental health difficulties, and microtransaction use. In addition, loot box use was associated with problematic video game use but not with problem gambling symptoms.

These findings contribute to the growing body of evidence suggesting that behavioural addictions and adolescent mental health are closely related. Although causal inferences cannot be drawn from the present cross-sectional study, the results highlight the importance of monitoring both psychological well-being and digital spending behaviours in adolescent populations. Future longitudinal studies are needed to clarify the direction and mechanisms underlying these associations.

## Figures and Tables

**Table 1:** Sample characteristics.

Characteristic	*N* = 343 ^1^
Total score on SOGS-RA (gambling)	0.83 (1.48)
Total score on IGDS9-SF (gaming)	13.2 (4.6)
Gender	
Female	129 (38%)
Male	208 (61%)
Prefer not to say	6 (1.7%)
Age	14.24 (1.49)
Opened loot box in the last year	
No	269 (78%)
Yes	74 (22%)
Microtransactions in the last year	
No	223 (65%)
Yes	120 (35%)
Total SDQ score	10.1 (5.3)

^1^ Mean (*SD*); *n* (*%*).

**Table 2:** Summary statistics for outcome variables.

	*n*	Mean	*SD*	Variance	Median	Q1	Q3
Total score on IGDS9-SF (gaming)	343	13.22	4.58	21.00	12	10	15
Total score on SOGS-RA (gambling)	343	0.83	1.48	2.19	0	0	1

**Table 3:** Risk markers.

Risk Marker	Description
Total SDQ score	The SDQ total score can range from 0 to 40; Cronbach’s α = 0.78, 95% confidence interval: 0.74–0.81)
Gender	Female (Reference level) Male Prefer not to say
Age	Numerical age
Opened loot box in last year	No (Reference level) Yes
Microtransactions in last year	No (Reference level) Yes

**Table 4:** Regression models: IGDS9-SF (Poisson) and SOGS-RA (Negative Binomial).

	Total Score on IGDS9-SF (Gaming)—Poisson Model				Total Score on SOGS-RA (Gambling)—Negative Binomial Model			
Risk Markers	Incidence Rate Ratios	CI	Statistic	*p*	Incidence Rate Ratios	CI	Statistic	*p*
(Intercept)	12.381	9.303–16.467	17.274	<0.001	0.438	0.084–2.263	−0.972	0.331
Gender: Male	1.208	1.126–1.298	5.218	<0.001	2.890	1.846–4.590	4.642	<0.001
Gender: Prefer not to say	1.120	0.897–1.381	1.029	0.303	3.046	0.990–10.056	1.945	0.052
Age	0.974	0.955–0.994	−2.580	0.010	0.903	0.803–1.014	−1.746	0.081
Loot boxes: Yes	1.130	1.052–1.213	3.361	0.001	1.061	0.703–1.595	0.284	0.776
Microtransactions: Yes	1.166	1.091–1.246	4.520	<0.001	1.741	1.193–2.543	2.903	0.004
Total SDQ score	1.022	1.016–1.028	7.412	<0.001	1.095	1.059–1.134	5.330	<0.001
Observations	343				343			
R^2^ Nagelkerke	0.546				0.337			
Deviance	302.253				295.676			
AIC	1819.922				792.447			
log-Likelihood	−902.961				−388.223			

## Data Availability

The data presented in this study are available on request from the corresponding author due to the inclusion of information from Spanish minors, whose disclosure is restricted for legal and data protection reasons.

## Supplementary Materials

The following supporting information can be downloaded at: https://www.mdpi.com/article/10.3390/healthcare14131846/s1.

## Abbreviations

The following abbreviations are used in this manuscript:

IGD	Internet Gaming Disorder
SDQ	Strengths and Difficulties Questionnaire
SOGS-RA	South Oaks Gambling Screen—Revised for Adolescents
