# Supplementary material for: Internet Gaming Disorder, Problem Gambling Symptoms and Mental Health in Spanish Adolescents: A Cross-Sectional Study on the Role of Microtransactions and Loot Boxes
Source: Healthcare (Basel). 2026 Jun 25;14(13):1846. doi: 10.3390/healthcare14131846 (PMC13360710; doi:10.3390/healthcare14131846)
Supplement: Supplementary file 1 [file healthcare-14-01846-s001.zip › healthcare-4347917-supplementary.pdf]

Supplementary Materials compares the characteristics of the final and original samples. The Wilcoxon rank sum test was used to test for significant differences (alpha = 0.05) for continuous variables, and Pearson's Chi-squared test for categorical variables (alpha = 0.05). Significant differences were identified for gender: the original sample was skewed towards females (59%), but the final sample, following listwise deletion, is skewed toward males (61%). The SDQ score for emotional symptoms was significantly lower in the final sample (mean = 2.71 [standard deviation = 2.26]), compared to the original sample (3.17 [2.25]).

Table S1. Comparison of final and original sample across outcome and predictor variables

Characteristic	Excluded Cases N = 507[1]	Included Cases N = 343[1]	p-value[2]
Total score on SOGS-RA (gambling)	1.87 (3.47)	0.83 (1.48)	0.4
Missing	476	0	
Total score on IGDS9-SF (gaming)	13.1 (4.3)	13.2 (4.6)	>0.9
Missing	203	0	
Gender			<0.001
Female	301 (59%)	129 (38%)	
Male	196 (39%)	208 (61%)	
Prefer not to say	10 (2.0%)	6 (1.7%)	
Age	14.30 (1.44)	14.24 (1.49)	0.5

Table S1. Comparison of final and original sample across outcome and predictor variables

Opened loot box in the last year			0.5
No	258 (76%)	269 (78%)	
Yes	80 (24%)	74 (22%)	
Missing	169	0	
Microtransactions in the last year			0.2
No	234 (69%)	223 (65%)	
Yes	104 (31%)	120 (35%)	
Missing	169	0	
SDQ total score	10.6 (5.7)	10.1 (5.3)	0.3

[1] Mean (SD); n (%)

[2] Wilcoxon rank sum test; Pearson's Chi-squared test
